# Stereotactic radiosurgery (SRS) for brain metastases: a systematic review

**DOI:** 10.1186/1748-717X-9-155

**Published:** 2014-07-12

**Authors:** Carsten Nieder, Anca L Grosu, Laurie E Gaspar

**Affiliations:** 1 Department of Oncology and Palliative Medicine, Nordland Hospital, 8092 Bodø, Norway; 2 Department of Radiation Oncology, University Hospital Freiburg, 79106 Freiburg, Germany; 3 Department of Radiation Oncology, University of Colorado, 80045 Aurora, CO, USA

**Keywords:** Brain metastases, Radiation treatment, Radiotherapy, Stereotactic radiosurgery

## Abstract

In many patients with brain metastases, the primary therapeutic aim is symptom palliation and maintenance of neurologic function, but in a subgroup, long-term survival is possible. Local control in the brain, and absent or controlled extracranial sites of disease are prerequisites for favorable survival. Stereotactic radiosurgery (SRS) is a focal, highly precise treatment option with a long track record. Its clinical development and implementation by several pioneering institutions eventually rendered possible cooperative group randomized trials. A systematic review of those studies and other landmark studies was undertaken. Most clinicians are aware of the potential benefits of SRS such as a short treatment time, a high probability of treated-lesion control and, when adhering to typical dose/volume recommendations, a low normal tissue complication probability. However, SRS as sole first-line treatment carries a risk of failure in non-treated brain regions, which has resulted in controversy around when to add whole-brain radiotherapy (WBRT). SRS might also be prescribed as salvage treatment in patients relapsing despite previous SRS and/or WBRT. An optimal balance between intracranial control and side effects requires continued research efforts.

## 

Historically, treatment options for patients with brain metastases from solid tumors were limited to surgery and/or whole-brain radiotherapy (WBRT) supported by corticosteroids if indicated, with few systemic therapies available that were able to impact on extracranial metastases. Therefore, median survival after non-surgical management typically was in the range of 3-4 months [1]. Both intra- and extracranial disease progression was common, resulting in complex clinical situations with components of decreasing performance status (PS), neurological status, and impaired organ function, e.g. from liver and lung metastases. Few patients who relapsed in the brain received salvage treatment such as reirradiation [2,3]. Partial brain fields or repeat WBRT were available but the efficacy of these salvage attempts was modest [4]. There was an urgent need for new treatment approaches, and among several avenues of research, clinical implementation of single fraction stereotactic radiosurgery (SRS) during the 1980s probably was the single most important innovation. The neurosurgery field gets credit for the early exploration of SRS [5-7], but the evolution of imaging, radiation treatment planning and delivery technology had to occur before several dedicated research groups were able to safely deliver SRS to patients with brain metastases [8-11]. The present review, which is based on a well defined method for identification of relevant studies, focuses on recent developments.

## Methods

A systematic search of the citation database Scopus (Elsevier B.V., http://www.scopus.com) by use of the terms ‘stereotactic AND brain’ and ‘radiosurgery’ was performed on 17^th^ January 2014. We did not use more specific search terms in order to avoid missing relevant publications. Articles were selected irrespective of language, year of publication and article type (review, guideline, clinical study, experimental study etc.), as described previously [12]. In order to determine whether or not a given article reported on SRS for brain metastases we accessed its abstract. Then, all articles dealing with the subject of this review were ranked by number of citations (field ‘times cited’ in the Scopus citation database) in order to create a list of articles with the highest number of citations, referred to as landmark studies (list available on request from the corresponding author). The top 50 articles were reviewed for contents, study type (phase I, II, III, retrospective etc.) and outcomes. Given that such hitlists are dominated by older publications because recent articles are less likely to have accumulated high numbers of citations [13], separate searches were performed that covered the years 2011 and 2012, respectively. The authors also used their reference lists from previous reviews [14-17] to cross-check for important studies that might not have been cited as often as expected. In addition, on 22^nd^ March 2014 the database Medline/PubMed was searched by the two terms mentioned above.

## Results and discussion

Table 1 contains the 25 most-cited articles [18-42]. These were published between 1990 and 2010. Of these, the prospective randomized clinical trials acquired the highest numbers of citations per year [18-20]. Most clinically relevant aspects of SRS for brain metastases are represented in the table, including the role of SRS alone compared to SRS plus WBRT, the role of WBRT alone compared to WBRT plus SRS, the role of SRS compared to surgery, patient selection based on prognostic models, SRS for recurrent disease, radiobiology of SRS, and toxicity. Issues related to the outcomes of different treatment planning approaches and treatment units ranked among the top 50 rather than the top 25 articles [10,43-66]. The most cited publications from the year 2011 did not cover any new aspects [67-71]. However the 2012 articles focused on new topics such as SRS to resection cavities in the postoperative setting, SRS in patients with more than 4 brain metastases, and new systemic treatments in addition to SRS [72-76]. Fractionated stereotactic radiotherapy was not among the most-cited topics.

**Table 1:** Articles with most citations (ranked by absolute count)

***Authors and year of publication***	***Study description***	***Absolute citation count***	***Citations per year***
Andrews et al. 2004 [18]	RTOG 9508 randomised trial	812	81
Aoyama et al. 2006 [19]	SRS ± WBRT randomised trial	527	66
Kondziolka et al. 1999 [20]	WBRT ± SRS randomised trial	517	37
Flickinger et al. 1994 [21]	SRS for solitary BM, multi institutional	445	22
Shaw et al. 2000 [22]	RTOG protocol 90-05	393	28
Alexander et al. 1995 [23]	Retrospective study	377	21
Auchter et al. 1996 [24]	SRS for resectable single BM, multi institutional	344	20
Chang et al. 2009 [25]	SRS ± WBRT randomised trial	314	63
Sneed et al. 2002 [26]	SRS ± WBRT, multi institutional	286	26
Sneed et al. 1999 [27]	SRS ± WBRT, single institution	286	20
Pirzkall et al. 1998 [28]	SRS ± WBRT, single institution	229	15
Sperduto et al. 2008 [29]	Prognostic score, incl. RTOG 95-08 data	216	36
Mehta et al. 1992 [30]	Prospective single arm, n = 40	186	9
Bindal et al. 1996 [31]	SRS vs. resection	182	10
Engenhart et al. 1993 [32]	Retrospective study	178	9
Mori et al. 1998 [33]	SRS for melanoma BM	169	11
Hall & Brenner 1993 [34]	Radiobiology of SRS	169	8
Shiau et al. 1997 [35]	Local control after SRS	165	10
Aoyama et al. 2007 [36]	Neurocognitive outcome, randomised trial	163	23
Muacevic et al. 1999 [37]	SRS vs. resection	163	11
Chao et al. 2001 [38]	Radionecrosis vs. relapse after SRS	161	12
Adler et al. 1992 [39]	Retrospective study	161	8
Sanghavi et al. 2001 [40]	Multi institutional, stratified for prognosis	156	12
Sperduto et al. 2010 [41]	Prognostic score, diagnosis specific	155	39
O’Neill et al. 2003 [42]	SRS vs. resection	155	14

RTOG: Radiation Therapy Oncology Group, SRS: stereotactic radiosurgery, WBRT: whole-brain radiotherapy, BM: brain metastases.

We will not discuss postoperative SRS in great detail, because this investigational approach so far is not yet supported by randomized trials [76]. Phase 2 data suggests that postoperative SRS is associated with high rates of local control, especially for non-superficial brain metastases of limited size (<3 cm) [77]. Metastases ≥3 cm with superficial dural/pial involvement demonstrated the highest risk of local failure. In a different study, patients with breast cancer had the highest risk of leptomeningeal progression following SRS (24% at 1 year) [78]. Head to head comparisons of SRS to other types of postoperative radiotherapy, including WBRT, are needed to conclusively evaluate the pros and cons of SRS in this setting.

Some institutions have more than 25 years of experience with SRS and have collected data on thousands of patients, often transfered to multi institutional databases. A recent Japanese multi institutional prospective study included 1194 patients (76% with lung cancer) [79]. Its aim was to examine whether survival after SRS without WBRT as initial treatment for patients with 5-10 brain metastases (median 6) was non-inferior to that of patients with 2-4 lesions. Size limits were metastases <3 cm in longest diameter, largest tumor <10 ml in volume, and total cumulative volume ≤15 ml. Median survival was longest in patients with one lesion (n = 455, 13.9 months). But patients with 2-4 lesions had comparable survival to patients with 5-10 lesions (median survival 10.8 months, hazard ratio 0.97, 95% confidence interval 0.81-1.18). This met the pre-specified definition of non-inferiority, despite the development of new lesions in >60% of patients. Further salvage SRS was done in more than 40%, and 9% received salvage WBRT. The delivery of further SRS or WBRT was not significant different between the groups. Grade 3-4 adverse events occurred in up to 3% of patients in each group. Only 8% of patients died from their brain disease.

As displayed in Table 2, SRS achieves high rates of local progression-free survival of SRS-treated lesions and its efficacy may be less influenced by histology or radiosensitivity than that of fractionated radiotherapy [63]. At the same time, severe complications are observed in a minority of patients. This is the primary reason for many institutions to expand its utilization beyond the initial target population, which often was defined as those with 1-3 lesions. The Japanese study [79] suggests that SRS is reasonable for patients with up to 10 lesions. However, it is also known that number of initial lesions predicts the risk for development of new metastases and thus need for salvage treatment (new course of SRS or WBRT). Primary tumor type and extracranial disease status also impact on distant brain failure risk. Based on these three predictors, a nomogram was recently developed (n = 464 patients) [80]. Table 3 shows examples calculated according to this model, which should be validated further.

**Table 2:** Results of stereotactic radiosurgery (SRS) for brain metastases

**Reference**	***n *** **(patients/lesions)**	Prescribed dose (median; range [Gy])*	**Median OS (m)**	**1-year PFS (%)**
Pirzkall et al. 1998 [28]	236/311	20; 10-30	5.5	89
Cho et al. 1998 [81]	73/136	17.5; 6-50	7.8	80
Sneed et al. 1999 [27]	62/118^a^	18; 15-22	11.3	80
	43/117^b^	17.5; 15-22	11.1	86
Varlotto et al. 2003 [82]	137/208	16; 12-25	Not given	90
Andrews et al. 2004 [18]	164/269^c^	Not given; 15-24	6.5	82
Bhatnagar et al. 2006 [83]	205/4-18 lesions each^d^	16; 12-20	8.0	71

OS: overall survival in months; PFS: progression-free survival.

*Prescription isodose or point varied, some series included SRS plus WBRT.

^a^SRS only.

^b^SRS plus WBRT (no significant difference in OS and PFS between both groups).

^c^SRS plus WBRT.

^d^SRS plus/minus WBRT.

**Table 3:** **Estimates of 6-month survival without distant brain failure based on a new nomogram**[80]

**Primary tumor type**	**1-3 BM, stable systemic disease**	**1-3 BM, progressive systemic disease**	**4-13 BM, stable systemic disease**	**4-13 BM, progressive systemic disease**
Renal cell cancer	69%	67%	48%	46%
Malignant melanoma	57%	55%	32%	30%
Lung, adeno ca	74%	72%	55%	53%
Lung, squamous ca	58%	57%	33%	32%
Breast, Her-2 positive	73%	72%	53%	52%
Breast, Her-2 negative	67%	66%	43%	42%

Sex, age and race impact slightly on failure risk. The examples refer to approximately 55-60 years-old Caucasian females. The differences for male patients are in the order of 1-2%.

BM: brain metastases.

Post-SRS adjuvant WBRT reduces intracranial relapses and neurologic deaths but fails to improve the duration of functional independence and overall survival [67,84]. The major obstacle against the general adoption of combined SRS and WBRT is the fear of neurocognitive decline after WBRT. Patients with limited survival expectation because of progressive, uncontrollable extracranial disease should be managed with WBRT or best supportive care rather than SRS [85,86].

### First line SRS and the controversy around whole-brain radiotherapy

Focal treatment such as SRS, improves the local control observed with WBRT. In a small randomized study primarily addressing this endpoint, patients with 2-4 brain metastases (all ≤25 mm diameter) either received WBRT alone (30 Gy in 12 fractions) or WBRT plus SRS [20]. The study was stopped at an interim evaluation following accrual of just 27 patients. The rate of local failure at 1 year was 100% after WBRT alone but only 8% in patients who had boost SRS. In exploratory analysis, patients who received WBRT alone lived a median of 7.5 months, while those who received WBRT plus RS lived 11 months (p = 0.22). With only 27 patients, possible survival differences cannot be adequately assessed. A different randomized study by the Radiation Therapy Oncology Group (RTOG) enrolled 333 patients with 1-3 brain metastases [18]. Maximum diameter of the largest lesion was 4 cm and additional lesions could not exceed 3 cm. Minimum Karnofsky PS was 70. WBRT dose was 37.5 Gy in 15 fractions in both groups. SRS boost dose was adjusted to lesion size (24 Gy, 18 Gy and 15 Gy for lesions < 2 cm, > 2 cm but less than 3 cm, and > 3 cm but < 4 cm, respectively). Median survival was significantly better after SRS boost in patients with single brain metastasis. By multivariate analysis, survival was also improved in recursive partitioning analysis (RPA) class I patients (RPA details are provided in Table 4). SRS-treated patients were more likely to have a stable or improved PS at 6 months (43% vs 27%, p = 0.03). Central imaging review showed higher response rates at 3 months and better control of the treated lesions at 1 year, p = 0.01. The risk of developing a local recurrence was 43% greater with WBRT alone. The addition of temozolomide or erlotinib did not improve survival after WBRT and SRS in another randomized RTOG trial, which included only patients with non-small cell lung cancer [87].

**Table 4:** Prognostic value of recursive partitioning analysis (RPA) classes

**Reference**	**Number of patients**	**RPA class I**	**RPA class II**	**RPA class III**
Gaspar et al. 1997 [88]	1200	7.1	4.2	2.3
Lorenzoni et al. 2004 [89]	110 (SRS)	27.6	10.7	2.8
Franzin et al. 2009 [90]	185 (SRS)	17.0	10.0	3.0
Likhacheva et al. 2012 [91]	251 (SRS)	38.8	9.4	2.8
Zindler et al. 2013 [92]	380 (SRS)	18.0*	10.0*	4.0*
Sneed et al.	268 (SRS)	14.0	8.2	5.3
2002 [26]	301 (SRS + WBRT)	15.2	7.0	5.5

Median survival in months from different publications.

RPA class I: age <65 years, Karnofsky performance status ≥70, controlled primary tumor, no extracranial metastases.

RPA class II: all other patients.

RPA class III: Karnofsky performance status <70.

SRS: stereotactic radiosurgery, WBRT: whole-brain radiotherapy.

*estimated from Kaplan-Meier graphs included in the publication.

After many years of controversy about the role of combining WBRT with SRS and considerable variation in practice [27,28], a Japanese group completed a prospective randomized multicenter phase III study of SRS alone vs combined SRS and WBRT [19]. The primary endpoint was survival with an expected difference of 30%. The trial included adult patients with Karnofsky PS >60 and a maximum of 4 brain metastases, none exceeding 3 cm diameter. The patients were stratified by number of lesions (1 vs 2-4), extracranial tumor activity (activ vs stable, i.e. controlled for at least 6 months), and primary tumor (lung cancer vs others). WBRT was given in 10 fractions of 3 Gy. SRS dose varied with size of the lesion (up to 2 cm: 22-25 Gy, > 2 cm: 18-20 Gy margin dose), and was reduced by 30% if WBRT was given. The mean dose was 21.9 Gy in the SRS alone arm and 16.6 Gy in the combined arm. The combined arm contained 65 patients, the SRS arm 67 patients. Almost 50% of patients had a single lesion. The SRS group contained slightly more patients with PS 90-100% (66 vs 52%) and patients without neurological symptoms (70 vs 59%). However, the differences were not statistically significant. Median survival was 7.5 months after SRS plus WBRT and 8 months after SRS alone. One-year survival in the combined treatment arm was increased by 36%, but was not statistically significantly different, possibly due to low patient numbers (38.5 vs 28.4%, p > 0.05). There was no significant difference in the percentage of patients that died from predominantly neurologic causes (23 vs 19%). Age, PS, extracranial disease activity and status of the primary tumor were significant prognostic factors on multivariate analysis. After SRS alone, 2 patients developed serious late complications (radionecrosis and grade 4 seizures, respectively). After SRS plus WBRT, 3 patients developed a radionecrosis and 3 signs of leukencephalopathy. The rate of actuarial failure at 1 year was 47% after combined treatment, but significantly greater at 76% after SRS alone (relative increase of 62%; p < 0.001). New lesions developed in 42 vs 64% (p = 0.003). The risk was significantly higher in patients presenting with 2-4 lesions before treatment, those with active extracranial metastases and those with PS 70-80. WBRT reduced the risk of failure at the site of SRS from 27% to 11% after one year (p = 0.002).

The highly cited randomized trial from the M.D. Anderson Cancer Center re-emphasized patient selection issues as critical for overall survival [25]. In this trial, patients with 1-3 newly diagnosed brain metastases were randomly assigned to SRS plus WBRT or SRS alone, and over an almost 7-year time frame, 58 patients were recruited and stratified by RPA class, number of brain metastases, and histology. The primary endpoint was neurocognitive function: measured as a 5-point drop compared with baseline in the Hopkins Verbal Learning Test–Revised (HVLT-R) total recall at 4 months. An interim analysis showed that there was a high probability (96%) that patients assigned to receive SRS plus WBRT were more likely to show a decline in learning and memory function at 4 months than patients assigned to receive SRS alone. Further, at 4 months there were four deaths (13%) in the group that received SRS alone, and eight deaths (29%) in the group that received SRS plus WBRT, and 73% of patients in the SRS plus WBRT group were free from CNS recurrence at 1 year, compared with 27% of patients who received SRS alone (p = 0.0003). These differences in early death bring into question the generalizability of the HVLT-R score results. It is well known that a general disease-related decline due to progression, especially in the pre-terminal phase will cause a significant drop in neurocognitive function, and its attribution to a single component, such as WBRT can be misleading. Another potential confounder in evaluating survival following SRS, with or without WBRT, is the initiation of systemic treatment after radiation. Moreover, several drug regimens are known to impact on brain function [93]. Ongoing studies evaluate hippocampal sparing WBRT, which aims at reducing dose to critical structures and thereby risk of function decline, while maintaining improved brain control [94,95]. In many clinical scenarios, acceptable approaches include SRS or SRS plus WBRT, as also summarized in an American Society for Radiation Oncology (ASTRO) guideline [14] and recent reviews [84,96].

### Salvage SRS as reirradiation after whole-brain radiotherapy

The potential advantages of SRS as salvage treatment after WBRT were realized early during the development of SRS [97]. The RTOG completed a prospective phase 1 clinical trial (RTOG 90-05) of SRS in recurrent, previously irradiated primary brain tumors and brain metastases. This was a dose escalation trial, which included 100 patients with brain metastases after prior WBRT to a median dose of 30 Gy [22]. Eligible patients had received first-line radiotherapy at least 3 months prior to study entry, and in the study, the actual median interval was 17 months. Life expectancy was ≥3 months. Seventy-eight percent of patients had single lesions. Dose was determined by the maximum diameter of the tumor. Initial doses were 18 Gy for lesions ≤20 mm, 15 Gy for lesions measuring 21-30 mm, and 12 Gy for lesions measuring 31-40 mm. Dose was prescribed to the 50-90% isodose line, which was to encompass the entire enhancing target volume. The dose was escalated in 3 Gy increments providing there was not an excess of unacceptable toxicity. The trial eventually defined the maximum acutely tolerable SRS dose in this setting, except for lesions ≤20 mm where the dose was not escalated beyond 24 Gy because of investigators’ reluctance. While small lesions ≤20 mm can be treated with up to 24 Gy to the margin of the lesion, those that measure between 21 and 30 mm might receive 18 Gy, and those that measure between 31 and 40 mm 15 Gy. Median survival was 7.5 months. Long-term toxicity data for 64 brain metastases patients revealed four patients developed radionecrosis requiring operation 5-14 months after SRS. This study therefore provides tentative evidence that retreatment with SRS can produce extended survival, but the incidence of necrosis must be factored in.

More recent data were derived from a retrospective review of 106 patients irradiated for a median of 2 metastases (range, 1-12) with a median dose of 21 Gy (range, 12-24) prescribed to the 50% isodose [98]. With a median follow-up of 10.5 months, local control was 83% at 6 months and 60% at 1 year. Median progression-free survival was 6.2 months. Median overall survival was 11.7 months from salvage SRS, and 22 months from initial diagnosis. Comparable outcomes were achieved in another retrospective series that included 111 patients [99]. SRS doses were usually prescribed according to the RTOG 90-05 guidelines. Median survival was 9.9 months. Twenty-five percent of patients developed further local progression in spite of salvage SRS. Poorer local control was observed in lesions >2 cm, which usually had been treated with lower radiation doses. Caballero et al. analyzed 310 patients [100]. The median number of brain metastases was 3, and interval from WBRT to SRS 8 months. The median survival was 8.4 months overall and 12.0 vs. 7.9 months for single vs. multiple lesions (p = 0.001). There was no relationship between number of lesions and survival after excluding patients with single metastases. Retrospective population-based data from Canada suggested that salvage SRS after WBRT was not associated with compromised survival compared to immediate boost SRS [101]. Only preliminary experience with small numbers of patients exists for salvage SRS after first-line SRS [102]. No definitive conclusions regarding long-term safety have yet been published.

### Prognostic staging systems

Patients with brain metastases have always presented with a variable spectrum of number, size and location of lesions, with different pattern and activity of extracranial disease, and with a wide range of comorbidities and PS. Estimation of prognosis is possible by using developed staging systems (Table 4) [41,43,88,89]. For example the RTOG developed the first RPA to define 3 classes of patients with statistically different predicted survival [88]. Other systems for predicting survival include the score index for radiosurgery (SIR) [43], basic score for brain metastases (BSBM) [89], and diagnosis-specific graded prognostic assessment (DS-GPA) score [41]. The latter is an increasingly used 4-tiered system that provides survival outcomes for patients with lung, breast, kidney and gastrointestinal cancers as well as malignant melanoma. These patients were treated with a variety of different approaches including but not limited to SRS. Their data were also used to create a prognostic nomogram [103]. In a recent study, symptomatic patients had an increased hazard for all-cause mortality (hazard ratio, 1.4) and were more likely to experience neurologic death after SRS (42% vs 20%, p < 0 .0001) [104]. Relative to asymptomatic patients, symptomatic patients required more craniotomies (43% vs 5%, p < 0.0001) and were more likely to have RTOG grade 3 and 4 post-treatment symptoms (24% vs 5%; p < 0.0001).

## Conclusions

SRS results in a high probability of treated-lesion control and, when adhering to typical dose/volume recommendations, a low normal tissue complication probability. However, SRS as sole first-line treatment carries a risk of failure in non-treated brain regions. SRS might also be prescribed as salvage treatment in patients relapsing despite previous SRS and/or WBRT. An optimal balance between intracranial control and side effects requires continued research efforts. Such efforts are also necessary to integrate new systemic treatments and/or stereotactic body radiotherapy, which aim at prolonged extracranial disease control.

## Competing interest

The authors declare that they have no competing interests.

## Authors’ contributions

CN participated in the design of the study and extraction of the references. All authors helped to review the references and draft the manuscript. All authors read and approved the final manuscript.

## References

[B1] Nieder C Spanne O Mehta MP Grosu AL Geinitz H Presentation, patterns of care, and survival in patients with brain metastases: what has changed in the last 20 years? Cancer 2011 117 2505 2512 24048799 10.1002/cncr.25707

[B2] Shehata WM Hendrickson FR Hindo WA Rapid fractionation technique and re-treatment of cerebral metastases by irradiation Cancer 1974 34 257 261 4137158 10.1002/1097-0142(197408)34:2<257::aid-cncr2820340206>3.0.co;2-6

[B3] Kurup P Reddy S Hendrickson FR Results of re-irradiation for cerebral metastases Cancer 1980 46 2587 2589 7448697 10.1002/1097-0142(19801215)46:12<2587::aid-cncr2820461209>3.0.co;2-4

[B4] Nieder C Milas L Ang KK Tissue tolerance to reirradiation Semin Radiat Oncol 2000 10 200 209 11034631 10.1053/srao.2000.6593

[B5] Leksell L The stereotactic method and radiosurgery of the brain Acta Chir Scan 1951 102 316 319 14914373

[B6] Gutin PH Bernstein M Stereotactic interstitial brachytherapy for malignant brain tumors Prog Exp Tumor Res 1984 28 166 182 6484199 10.1159/000408244

[B7] Leksell DG Stereotactic radiosurgery. Present status and future trends Neurol Res 1987 9 60 68 2886946 10.1080/01616412.1987.11739775

[B8] Colombo F Benedetti A Pozza F Zanardo A Avanzo RC Chierego G Marchetti C Stereotactic radiosurgery utilizing a linear accelerator Appl Neurophysiol 1985 48 133 145 3915642 10.1159/000101117

[B9] Greitz T Lax I Bergström M Arndt J Berggren BM Blomgren H Boëthius J Lindqvist M Ribbe T Steiner L Stereotactic radiation therapy of intracranial lesions Methodologic aspects Acta Radiol Oncol 1986 25 81 89 10.3109/02841868609136383 3012962

[B10] Sturm V Kober B Höver KH Schlegel W Boesecke R Pastyr O Hartmann GH Schabbert S zum Winkel K Kunze S Stereotactic percutaneous single dose irradiation of brain metastases with a linear accelerator Int J Radiat Oncol Biol Phys 1987 13 279 282 3102416 10.1016/0360-3016(87)90140-4

[B11] Lutz W Winston KR Maleki N A system for stereotactic radiosurgery with a linear accelerator Int J Radiat Oncol Biol Phys 1988 14 373 381 3276655 10.1016/0360-3016(88)90446-4

[B12] Nieder C Astner ST Grosu AL Glioblastoma research 2006-2010: pattern of citation and systematic review of highly cited articles Clin Neurol Neurosurg 2012 114 1207 1210 22516416 10.1016/j.clineuro.2012.03.049

[B13] Nieder C Andratschke NH Grosu AL Increasing frequency of reirradiation studies in radiation oncology: systematic review of highly cited articles Am J Cancer Res 2013 3 152 158 23593538 PMC3623835

[B14] Tsao MN Rades D Wirth A Lo SS Danielson BL Gaspar LE Sperduto PW Vogelbaum MA Radawski JD Wang JZ Gillin MT Mohideen N Hahn CA Chang EL Radiotherapeutic and surgical management for newly diagnosed brain metastasis(es): An American Society for Radiation Oncology evidence-based guideline Pract Radiat Oncol 2012 2 210 225 10.1016/j.prro.2011.12.004 PMC3808749 25925626

[B15] Patel SH Robbins JR Gore EM Bradley JD Gaspar LE Germano I Ghafoori P Henderson MA Lutz ST McDermott MW Patchell RA Robins HI Vassil AD Wippold FJ 2nd Videtic GM ACR Appropriateness Criteria: follow-up and retreatment of brain metastases Am J Clin Oncol 2012 35 302 306 22609733 10.1097/COC.0b013e31824be246

[B16] Suh JH Videtic GM Aref AM Germano I Goldsmith BJ Imperato JP Marcus KJ McDermott MW McDonald MW Patchell RA Robins HI Rogers CL Wolfson AH Wippold FJ 2nd Gaspar LE ACR Appropriateness Criteria: single brain metastasis Curr Probl Cancer 2010 34 162 174 20541055 10.1016/j.currproblcancer.2010.04.003

[B17] Gaspar LE Mehta MP Patchell RA Burri SH Robinson PD Morris RE Ammirati M Andrews DW Asher AL Cobbs CS Kondziolka D Linskey ME Loeffler JS McDermott M Mikkelsen T Olson JJ Paleologos NA Ryken TC Kalkanis SN The role of whole brain radiation therapy in the management of newly diagnosed brain metastases: a systematic review and evidence-based clinical practice guideline J Neurooncol 2010 96 17 32 19960231 10.1007/s11060-009-0060-9 PMC2808517

[B18] Andrews DW Scott CB Sperduto PW Flanders AE Gaspar LE Schell MC Werner-Wasik M Demas W Ryu J Bahary JP Souhami L Rotman M Mehta MP Curran WJ Whole brain radiation therapy with or without stereotactic radiosurgery boost for patients with one to three brain metastases: Phase III results of the RTOG 9508 randomised trial Lancet 2004 363 1665 1672 Cited 812 times 15158627 10.1016/S0140-6736(04)16250-8

[B19] Aoyama H Shirato H Tago M Nakagawa K Toyoda T Hatano K Kenjyo M Oya N Hirota S Shioura H Kunieda E Inomata T Hayakawa K Katoh N Kobashi G Stereotactic radiosurgery plus whole-brain radiation therapy vs stereotactic radiosurgery alone for treatment of brain metastases: A randomized controlled trial JAMA 2006 295 2483 2491 Cited 527 times 16757720 10.1001/jama.295.21.2483

[B20] Kondziolka D Patel A Lunsford LD Kassam A Flickinger JC Stereotactic radiosurgery plus whole brain radiotherapy versus radiotherapy alone for patients with multiple brain metastases Int J Radiat Oncol Biol Phys 1999 45 427 434 Cited 517 times 10487566 10.1016/s0360-3016(99)00198-4

[B21] Flickinger JC Kondziolka D Lunsford LD Coffey RJ Goodman ML Shaw EG Hudgins RW Weiner R Harsh GR Sneed PK Larson DA A multi-institutional experience with stereotactic radiosurgery for solitary brain metastasis Int J Radiat Oncol Biol Phys 1994 28 797 802 Cited 445 times 8138431 10.1016/0360-3016(94)90098-1

[B22] Shaw E Scott C Souhami L Dinapoli R Kline R Loeffler J Farnan N Single dose radiosurgical treatment of recurrent previously irradiated primary brain tumors and brain metastases: Final report of RTOG protocol 90-05 Int J Radiat Oncol Biol Phys 2000 47 291 298 Cited 393 times 10802351 10.1016/s0360-3016(99)00507-6

[B23] Alexander E III Moriarty TM Davis RB Wen PY Fine HA Black PM Kooy HM Loeffler JS Stereotactic radiosurgery for the definitive, noninvasive treatment of brain metastases J Natl Cancer Inst 1995 87 34 40 Cited 377 times 7666461 10.1093/jnci/87.1.34

[B24] Auchter RM Lamond JP Alexander E III Buatti JM Chappell R Friedman WA Kinsella TJ Levin AB Noyes WR Schultz CJ Loeffler JS Mehta MP A multiinstitutional outcome and prognostic factor analysis of radiosurgery for resectable single brain metastasis Int J Radiat Oncol Biol Phys 1996 35 27 35 Cited 344 times 8641923 10.1016/s0360-3016(96)85008-5

[B25] Chang EL Wefel JS Hess KR Allen PK Lang FF Kornguth DG Arbuckle RB Swint JM Shiu AS Maor MH Meyers CA Neurocognition in patients with brain metastases treated with radiosurgery or radiosurgery plus whole-brain irradiation: a randomised controlled trial Lancet Oncol 2009 10 1037 1044 Cited 314 times 19801201 10.1016/S1470-2045(09)70263-3

[B26] Sneed PK Suh JH Goetsch SJ Sanghavi SN Chappell R Buatti JM Regine WF Weltman E King VJ Breneman JC Sperduto PW Mehta MP A multi-institutional review of radiosurgery alone vs. radiosurgery with whole brain radiotherapy as the initial management of brain metastases Int J Radiat Oncol Biol Phys 2002 53 519 526 Cited 286 times 12062592 10.1016/s0360-3016(02)02770-0

[B27] Sneed PK Lamborn KR Forstner JM McDermott MW Chang S Park E Gutin PH Phillips TL Wara WM Larson DA Radiosurgery for brain metastases: Is whole brain radiotherapy necessary? Int J Radiat Oncol Biol Phys 1999 43 549 558 Cited 286 times 10078636 10.1016/s0360-3016(98)00447-7

[B28] Pirzkall A Debus J Lohr F Fuss M Rhein B Radiosurgery alone or in combination with whole-brain radiotherapy for brain metastases J Clin Oncol 1998 16 3563 3569 Cited 229 times 9817276 10.1200/JCO.1998.16.11.3563

[B29] Sperduto PW Berkey B Gaspar LE Mehta M Curran W A new prognostic index and comparison to three other indices for patients with brain metastases: An analysis of 1,960 patients in the RTOG database Int J Radiat Oncol Biol Phys 2008 70 510 514 Cited 216 times 17931798 10.1016/j.ijrobp.2007.06.074

[B30] Mehta MP Rozental JM Levin AB Mackie TR Kubsad SS Gehring MA Kinsella TJ Defining the role of radiosurgery in the management of brain metastases Int J Radiat Oncol Biol Phys 1992 24 619 625 Cited 186 times 1429083 10.1016/0360-3016(92)90706-n

[B31] Bindal AK Bindal RK Hess KR Shiu A Hassenbusch SJ Shi WM Sawaya R Surgery versus radiosurgery in the treatment of brain metastasis J Neurosurg 1996 84 748 754 Cited 182 times 8622147 10.3171/jns.1996.84.5.0748

[B32] Engenhart R Kimmig BN Hover KH Wowra B Romahn J Lorenz WJ Van Kaick G Wannenmacher M Long-term follow-up for brain metastases treated by percutaneous stereotactic single high-dose irradiation Cancer 1993 71 1353 1361 Cited 178 times 8435811 10.1002/1097-0142(19930215)71:4<1353::aid-cncr2820710430>3.0.co;2-6

[B33] Mori Y Kondziolka D Flickinger JC Kirkwood JM Agarwala S Lunsford LD Stereotactic radiosurgery for cerebral metastatic melanoma: Factors affecting local disease control and survival Int J Radiat Oncol Biol Phys 1998 42 581 589 Cited 169 times 9806518 10.1016/s0360-3016(98)00272-7

[B34] Hall EJ Brenner DJ The radiobiology of radiosurgery: Rationale for different treatment regimes for AVMs and malignancies Int J Radiat Oncol Biol Phys 1993 25 381 385 Cited 169 times 8420891 10.1016/0360-3016(93)90367-5

[B35] Shiau CY Sneed PK Shu HK Lamborn KR McDermott MW Chang S Nowak P Petti PL Smith V Verhey LJ Ho M Park E Wara WM Gutin PH Larson DA Radiosurgery for brain metastases: Relationship of dose and pattern of enhancement to local control Int J Radiat Oncol Biol Phys 1997 37 375 383 Cited 165 times 9069310 10.1016/s0360-3016(96)00497-x

[B36] Aoyama H Tago M Kato N Toyoda T Kenjyo M Hirota S Shioura H Inomata T Kunieda E Hayakawa K Nakagawa K Kobashi G Shirato H Neurocognitive function of patients with brain metastasis who received either whole brain radiotherapy plus stereotactic radiosurgery or radiosurgery alone Int J Radiat Oncol Biol Phys 2007 68 1388 1395 Cited 163 times 17674975 10.1016/j.ijrobp.2007.03.048

[B37] Muacevic A Kreth FW Horstmann GA Schmid-Elsaesser R Wowra B Steiger HJ Reulen HJ Surgery and radiotherapy compared with gamma knife radiosurgery in the treatment of solitary cerebral metastases of small diameter J Neurosurg 1999 91 35 43 Cited 162 times 10389878 10.3171/jns.1999.91.1.0035

[B38] Chao ST Suh JH Raja S Lee SY Barnett G The sensitivity and specificity of FDG PET in distinguishing recurrent brain tumor from radionecrosis in patients treated with stereotactic radiosurgery Int J Cancer 2001 96 191 197 Cited 161 times 11410888 10.1002/ijc.1016

[B39] Adler JR Cox RS Kaplan I Martin DP Stereotactic radiosurgical treatment of brain metastases J Neurosurg 1992 76 444 449 Cited 161 times 1738025 10.3171/jns.1992.76.3.0444

[B40] Sanghavi SN Miranpuri SS Chappell R Buatti JM Sneed PK Suh JH Regine WF Weltman E King VJ Goetsch SJ Breneman JC Sperduto PW Scott C Mabanta S Mehta MP Radiosurgery for patients with brain metastases: A multi-institutional analysis, stratified by the RTOG recursive partitioning analysis method Int J Radiat Oncol Biol Phys 2001 51 426 434 Cited 156 times 11567817 10.1016/s0360-3016(01)01622-4

[B41] Sperduto PW Chao ST Sneed PK Luo X Suh J Roberge D Bhatt A Jensen AW Brown PD Shih H Kirkpatrick J Schwer A Gaspar LE Fiveash JB Chiang V Knisely J Sperduto CM Mehta M Diagnosis-specific prognostic factors, indexes, and treatment outcomes for patients with newly diagnosed brain metastases: A multi-institutional analysis of 4,259 patients Int J Radiat Oncol Biol Phys 2010 77 655 661 Cited 155 times 19942357 10.1016/j.ijrobp.2009.08.025

[B42] O’Neill BP Iturria NJ Link MJ Pollock BE Ballman KV O’Fallon JR A comparison of surgical resection and stereotactic radiosurgery in the treatment of solitary brain metastases Int J Radiat Oncol Biol Phys 2003 55 1169 1176 Cited 155 times 12654423 10.1016/s0360-3016(02)04379-1

[B43] Weltman E Salvajoli JV Brandt RA De Morais Hanriot R Prisco FE Cruz JC De Oliveira Borges SR Wajsbrot DB Radiosurgery for brain metastases: A score index for predicting prognosis Int J Radiat Oncol Biol Phys 2000 46 1155 1161 Cited 152 times 10725626 10.1016/s0360-3016(99)00549-0

[B44] Loeffler JS Kooy HM Wen PY Fine HA Cheng CW Mannarino EG Tsai JS Alexander E III The treatment of recurrent brain metastases with stereotactic radiosurgery J Clin Oncol 1990 8 276 582 Cited 151 times 10.1200/JCO.1990.8.4.576 2179476

[B45] Mehta MP Tsao MN Whelan TJ Morris DE Hayman JA Flickinger JC Mills M Rogers CL Souhami L The American Society for Therapeutic Radiology and Oncology (ASTRO) evidence-based review of the role of radiosurgery for brain metastases Int J Radiat Oncol Biol Phys 2005 63 37 46 Cited 146 times 16111570 10.1016/j.ijrobp.2005.05.023

[B46] Flickinger JC An integrated logistic formula for prediction of complications from radiosurgery Int J Radiat Oncol Biol Phys 1989 17 879 885 Cited 143 times 2777680 10.1016/0360-3016(89)90082-5

[B47] Mori Y Kondziolka D Flickinger JC Logan T Lunsford LD Stereotactic radiosurgery for brain metastasis from renal cell carcinoma Cancer 1998 83 344 353 Cited 137 times 9669818 10.1002/(sici)1097-0142(19980715)83:2<344::aid-cncr19>3.0.co;2-t

[B48] Chidel MA Suh JH Reddy CA Chao ST Lundbeck MF Barnett GH Application of recursive partitioning analysis and evaluation of the use of whole brain radiation among patients treated with stereotactic radiosurgery for newly diagnosed brain metastases Int J Radiat Oncol Biol Phys 2000 47 993 999 Cited 129 times 10863070 10.1016/s0360-3016(00)00527-7

[B49] Breneman JC Warnick RE Albright RE Kukiatinant N Shaw J Armin D Tew J Stereotactic radiosurgery for the treatment of brain metastases: Results of a single institution series Cancer 1997 79 551 557 Cited 127 times 9028367 10.1002/(sici)1097-0142(19970201)79:3<551::aid-cncr18>3.0.co;2-2

[B50] Shaw E Scott C Souhami L Dinapoli R Bahary JP Kline R Wharam M Schultz C Davey P Loeffler J Del Rowe J Marks L Fisher B Shin K Radiosurgery for the treatment of previously irradiated recurrent primary brain tumors and brain metastases: Initial report of Radiation Therapy Oncology Group protocol 90-05 Int J Radiat Oncol Biol Phys 1996 34 647 654 Cited 125 times 8621289 10.1016/0360-3016(95)02106-x

[B51] Hasegawa T Kondziolka D Flickinger JC Germanwala A Lunsford LD Adler JR Gutin PH Tabar V Piepmeier JM Petrovich Z Brain metastases treated wlth radiosurgery alone: An alternative to whole brain radiotherapy? Neurosurgery 2003 52 1318 1326 Cited 123 times 12762877 10.1227/01.neu.0000064569.18914.de

[B52] Regine WF Huhn JL Patchell RA St Clair WH Strottmann J Meigooni A Sanders M Young AB Risk of symptomatic brain tumor recurrence and neurologic deficit after radiosurgery alone in patients with newly diagnosed brain metastases: Results and implications Int J Radiat Oncol Biol Phys 2002 Cited 122 times 333 338 Cited 122 times 11872278 10.1016/s0360-3016(01)02645-1

[B53] Nedzi LA Kooy H Alexander E III Gelman RS Loeffler JS Variables associated with the development of complications from radiosurgery of intracranial tumors Int J Radiat Oncol Biol Phys 1991 21 591 599 Cited 121 times 1907957 10.1016/0360-3016(91)90675-t

[B54] Somaza S Kondziolka D Lunsford LD Kirkwood JM Flickinger JC Stereotactic radiosurgery for cerebral metastatic melanoma J Neurosurg 1993 79 661 666 Cited 120 times 8410244 10.3171/jns.1993.79.5.0661

[B55] Rutigliano MJ Lunsford LD Kondziolka D Strauss MJ Khanna V Green M Patterson RH Taub E Friedman WA Ciric I The cost effectiveness of stereotactic radiosurgery versus surgical resection in the treatment of solitary metastatic brain tumors Neurosurgery 1995 37 445 455 Cited 116 times 7501109 10.1227/00006123-199509000-00012

[B56] Sheehan JP Sun MH Kondziolka D Flickinger J Lunsford LD Radiosurgery in patients with renal cell carcinoma metastasis to the brain: Long-term outcomes and prognostic factors influencing survival and local tumor control J Neurosurg 2003 98 342 349 Cited 115 times 12593621 10.3171/jns.2003.98.2.0342

[B57] Lavine SD Petrovich Z Cohen-Gadol AA Masri LS Morton DL O’Day SJ Essner R Zelman V Yu C Luxton G Apuzzo ML Gamma knife radiosurgery for metastatic melanoma: An analysis of survival, outcome, and complications Neurosurgery 1999 44 59 66 Cited 114 times 9894964 10.1097/00006123-199901000-00031

[B58] Cardinale RM Benedict SH Wu Q Zwicker RD Gaballa HE Mohan R A comparison of three stereotactic radiotherapy techniques; arcs vs. noncoplanar fixed fields vs. intensity modulation Int J Radiat Oncol Biol Phys 1998 42 431 436 9788426 10.1016/s0360-3016(98)00206-5

[B59] Kooy HM Van Herk M Barnes PD Alexander E III Dunbar SF Tarbell NJ Mulkern RV Holupka EJ Loeffler JS Image fusion for stereotactic radiotherapy and radiosurgery treatment planning Int J Radiat Oncol Biol Phys 1994 28 1229 1234 Cited 112 times 8175410 10.1016/0360-3016(94)90499-5

[B60] Schöggl A Kitz K Reddy M Wolfsberger S Schneider B Dieckmann K Ungersböck K Defining the role of stereotactic radiosurgery versus microsurgery in the treatment of single brain metastases Acta Neurochir 2000 142 621 626 Cited 108 times 10949435 10.1007/s007010070104

[B61] Linskey ME Andrews DW Asher AL Burri SH Kondziolka D Robinson PD Ammirati M Cobbs CS Gaspar LE Loeffler JS McDermott M Mehta MP Mikkelsen T Olson JJ Paleologos NA Patchell RA Ryken TC Kalkanis SN The role of stereotactic radiosurgery in the management of patients with newly diagnosed brain metastases: A systematic review and evidence-based clinical practice guideline J Neurooncol 2010 96 45 68 Cited 103 times 19960227 10.1007/s11060-009-0073-4 PMC2808519

[B62] Petrovich Z Yu C Giannotta SL O’Day S Apuzzo ML Survival and pattern of failure in brain metastasis treated with stereotactic gamma knife radiosurgery J Neurosurg 2002 97 499 506 Cited 93 times 12507085 10.3171/jns.2002.97.supplement

[B63] Brown PD Brown CA Pollock BE Gorman DA Foote RL Loeffler JS Adler JR Ryu S Gutin PH Kondziolka D Piepmeier JM Stereotactic radiosurgery for patients with “radioresistant” brain metastases Neurosurgery 2002 51 656 667 Cited 91 times 12188943

[B64] Shirato H Takamura A Tomita M Suzuki K Nishioka T Isu T Kato T Sawamura Y Miyamachi K Hiroshi A Miyasaka K Stereotactic irradiation without whole-brain irradiation for single brain metastasis Int J Radiat Oncol Biol Phys 1997 37 385 391 Cited 91 times 9069311 10.1016/s0360-3016(96)00488-9

[B65] Coffey RJ Flickinger JC Bissonette DJ Lunsford LD Radiosurgery for solitary brain metastases using the cobalt-60 gamma unit: Methods and results in 24 patients Int J Radiat Oncol Biol Phys 1991 20 1287 1295 Cited 89 times 1646195 10.1016/0360-3016(91)90240-5

[B66] Nakamura JL Verhey LJ Smith V Petti PL Lamborn KR Larson DA Wara WM McDermott MW Sneed PK Dose conformity of Gamma Knife radiosurgery and risk factors for complications Int J Radiat Oncol Biol Phys 2001 51 1313 1319 Cited 88 times 11728692 10.1016/s0360-3016(01)01757-6

[B67] Kocher M Soffietti R Abacioglu U Villà S Fauchon F Baumert BG Fariselli L Tzuk-Shina T Kortmann RD Carrie C Ben Hassel M Kouri M Valeinis E van den Berge D Collette S Collette L Mueller RP J Clin Oncol 2011 29 134 141 Cited 210 times 21041710 10.1200/JCO.2010.30.1655 PMC3058272

[B68] Liew DN Kano H Kondziolka D Mathieu D Niranjan A Flickinger JC Kirkwood JM Tarhini A Moschos S Lunsford LD Outcome predictors of gamma knife surgery for melanoma brain metastases J Neurosurg 2011 114 769 779 Cited 29 times 20524829 10.3171/2010.5.JNS1014

[B69] Kondziolka D Kano H Harrison GL Yang HC Liew DN Niranjan A Brufsky AM Flickinger JC Lunsford LD Stereotactic radiosurgery as primary and salvage treatment for brain metastases from breast cancer J Neurosurg 2011 114 792 800 Cited 26 times 20887087 10.3171/2010.8.JNS10461

[B70] Jenkinson MD Haylock B Shenoy A Husband D Javadpour M Management of cerebral metastasis: Evidence-based approach for surgery, stereotactic radiosurgery and radiotherapy Eur J Cancer 2011 47 649 655 Cited 23 times 21196112 10.1016/j.ejca.2010.11.033

[B71] Minniti G Clarke E Lanzetta G Osti MF Trasimeni G Bozzao A Romano A Enrici RM Stereotactic radiosurgery for brain metastases: Analysis of outcome and risk of brain radionecrosis Radiat Oncol 2011 6 48 Cited 21 times 21575163 10.1186/1748-717X-6-48 PMC3108308

[B72] Tsao M Xu W Sahgal A A meta-analysis evaluating stereotactic radiosurgery, whole-brain radiotherapy, or both for patients presenting with a limited number of brain metastases Cancer 2012 118 2486 2493 Cited 14 times 21887683 10.1002/cncr.26515

[B73] Knisely JP Yu JB Flanigan J Sznol M Kluger HM Chiang VL Radiosurgery for melanoma brain metastases in the ipilimumab era and the possibility of longer survival J Neurosurg 2012 117 227 233 Cited 13 times 22702482 10.3171/2012.5.JNS111929 PMC6098938

[B74] Kelly PJ Lin YB Yu AY Alexander BM Hacker F Marcus KJ Weiss SE Stereotactic irradiation of the postoperative resection cavity for brain metastasis: A frameless linear accelerator-based case series and review of the technique Int J Radiat Oncol Biol Phys 2012 82 95 101 Cited 13 times 21168282 10.1016/j.ijrobp.2010.10.043

[B75] Hunter GK Suh JH Reuther AM Vogelbaum MA Barnett GH Angelov L Weil RJ Neyman G Chao ST Treatment of five or more brain metastases with stereotactic radiosurgery Int J Radiat Oncol Biol Phys 2012 83 1394 1398 Cited 10 times 22209150 10.1016/j.ijrobp.2011.10.026

[B76] Roberge D Parney I Brown PD Radiosurgery to the postoperative surgical cavity: Who needs evidence? Int J Radiat Oncol Biol Phys 2012 83 486 493 Cited 10 times 22099047 10.1016/j.ijrobp.2011.09.032

[B77] Brennan C Yang TJ Hilden P Zhang Z Chan K Yamada Y Chan TA Lymberis SC Narayana A Tabar V Gutin PH Ballangrud Å Lis E Beal K A phase 2 trial of stereotactic radiosurgery boost after surgical resection for brain metastases Int J Radiat Oncol Biol Phys 2014 88 130 136 24331659 10.1016/j.ijrobp.2013.09.051 PMC5736310

[B78] Atalar B Modlin LA Choi CY Adler JR Gibbs IC Chang SD Harsh GR 4th Li G Nagpal S Hanlon A Soltys SG Risk of leptomeningeal disease in patients treated with stereotactic radiosurgery targeting the postoperative resection cavity for brain metastases Int J Radiat Oncol Biol Phys 2013 87 713 718 24054875 10.1016/j.ijrobp.2013.07.034

[B79] Yamamoto M Serizawa T Shuto T Akabane A Higuchi Y Kawagishi J Yamanaka K Sato Y Jokura H Yomo S Nagano O Kenai H Moriki A Suzuki S Kida Y Iwai Y Hayashi M Onishi H Gondo M Sato M Akimitsu T Kubo K Kikuchi Y Shibasaki T Goto T Takanashi M Mori Y Takakura K Saeki N Kunieda E Aoyama H Stereotactic radiosurgery for patients with multiple brain metastases (JLGK0901): a multi-institutional prospective observational study Lancet Oncol 2014 S1470-2045(14)70061-0. doi: 10.1016/S1470-2045(14)70061-0 10.1016/S1470-2045(14)70061-0 24621620

[B80] Ayala-Peacock DN Peiffer AM Lucas JT Isom S Kuremsky JG Urbanic JJ Bourland JD Laxton AW Tatter SB Shaw EG Chan MD A nomogram for predicting distant brain failure in patients treated with gamma knife stereotactic radiosurgery without whole brain radiotherapy Neuro Oncol 2014 2014 2014 10.1093/neuonc/nou018 PMC4136890 24558022

[B81] Cho KH Hall WA Gerbi BJ Higgins PD Bohen M Clark HB Patient selection criteria for the treatment of brain metastases with stereotactic radiosurgery J Neurooncol 1998 40 73 86 9874189 10.1023/a:1006169109920

[B82] Varlotto JM Flickinger JC Niranjan A Bhatnagar AK Kondziolka D Lunsford LD Analysis of tumor control and toxicity in patients who have survived at least one year after radiosurgery for brain metastases Int J Radiat Oncol Biol Phys 2003 57 452 464 12957257 10.1016/s0360-3016(03)00568-6

[B83] Bhatnagar AK Flickinger JC Kondziolka D Lunsford LD Stereotactic radiosurgery for four or more intracranial metastases Int J Radiat Oncol Biol Phys 2006 64 898 903 Cited 86 times 16338097 10.1016/j.ijrobp.2005.08.035

[B84] Soon YY Tham IW Lim KH Koh WY Lu JJ Surgery or radiosurgery plus whole brain radiotherapy versus surgery or radiosurgery alone for brain metastases Cochrane Database Syst Rev 2014 3 CD009454 10.1002/14651858.CD009454.pub2 PMC6457788 24585087

[B85] Nieder C Norum J Dalhaug A Aandahl G Engljähringer K Best supportive care in patients with brain metastases and adverse prognostic factors: development of improved decision aids Support Care Cancer 2013 21 2671 2678 23686380 10.1007/s00520-013-1840-5

[B86] Nieder C Norum J Dalhaug A Aandahl G Pawinski A Radiotherapy versus best supportive care in patients with brain metastases and adverse prognostic factors Clin Exp Metastasis 2013 30 723 729 23392634 10.1007/s10585-013-9573-x

[B87] Sperduto PW Wang M Robins HI Schell MC Werner-Wasik M Komaki R Souhami L Buyyounouski MK Khuntia D Demas W Shah SA Nedzi LA Perry G Suh JH Mehta MP A phase 3 trial of whole brain radiation therapy and stereotactic radiosurgery alone versus WBRT and SRS with temozolomide or erlotinib for non-small cell lung cancer and 1 to 3 brain metastases: Radiation Therapy Oncology Group 0320 Int J Radiat Oncol Biol Phys 2013 85 1312 1318 23391814 10.1016/j.ijrobp.2012.11.042 PMC3740376

[B88] Gaspar L Scott C Rotman M Asbell S Phillips T Wasserman T McKenna WG Byhardt R Recursive partitioning analysis (RPA) of prognostic factors in three Radiation Therapy Oncology Group (RTOG) brain metastases trials Int J Radiat Oncol Biol Phys 1997 37 745 751 9128946 10.1016/s0360-3016(96)00619-0

[B89] Lorenzoni J Devriendt D Massager N David P Ruíz S Vanderlinden B Van Houtte P Brotchi J Levivier M Radiosurgery for treatment of brain metastases: estimation of patient eligibility using three stratification systems Int J Radiat Oncol Biol Phys 2004 60 218 224 15337559 10.1016/j.ijrobp.2004.02.017

[B90] Franzin A Snider S Picozzi P Bolognesi A Serra C Vimercati A Passarin O Mortini P Evaluation of different score index for predicting prognosis in gamma knife radiosurgical treatment for brain metastasis Int J Radiat Oncol Biol Phys 2009 74 707 713 19095375 10.1016/j.ijrobp.2008.08.062

[B91] Likhacheva A Pinnix CC Parikh N Allen PK Guha-Thakurta N McAleer M Sulman EP Mahajan A Shiu A Luo D Chiu M Brown PD Prabhu SS Chang EL Validation of Recursive Partitioning Analysis and Diagnosis-Specific Graded Prognostic Assessment in patients treated initially with radiosurgery alone J Neurosurg 2012 117 38 44 23205787 10.3171/2012.3.GKS1289 PMC3910154

[B92] Zindler JD Rodrigues G Haasbeek CJ De Haan PF Meijer OW Slotman BJ Lagerwaard FJ The clinical utility of prognostic scoring systems in patients with brain metastases treated with radiosurgery Radiother Oncol 2013 106 370 374 23522151 10.1016/j.radonc.2013.01.015

[B93] Dutta V Chemotherapy, neurotoxicity, and cognitive changes in breast cancer J Cancer Res Ther 2011 7 264 269 22044805 10.4103/0973-1482.87008

[B94] Oskan F Ganswindt U Schwarz SB Manapov F Belka C Niyazi M Hippocampus sparing in whole-brain radiotherapy: A review Strahlenther Onkol 2014 190 337 341 24452816 10.1007/s00066-013-0518-8

[B95] Prokic V Wiedenmann N Fels F Schmucker M Nieder C Grosu AL Whole brain irradiation with hippocampal sparing and dose escalation on multiple brain metastases: a planning study on treatment concepts Int J Radiat Oncol Biol Phys 2013 85 264 270 22516808 10.1016/j.ijrobp.2012.02.036

[B96] Patil CG Pricola K Sarmiento JM Garg SK Bryant A Black KL Whole brain radiation therapy (WBRT) alone versus WBRT and radiosurgery for the treatment of brain metastases Cochrane Database Syst Rev 2012 9 CD006121 10.1002/14651858.CD006121.pub3 PMC6457849 22972090

[B97] Ammirati M Cobbs CS Linskey ME Paleologos NA Ryken TC Burri SH Asher AL Loeffler JS Robinson PD Andrews DW Gaspar LE Kondziolka D McDermott M Mehta MP Mikkelsen T Olson JJ Patchell RA Kalkanis SN The role of retreatment in the management of recurrent/progressive brain metastases: a systematic review and evidence-based clinical practice guideline J Neurooncol 2010 96 85 96 19957016 10.1007/s11060-009-0055-6 PMC2808530

[B98] Kurtz G Zadeh G Gingras-Hill G Millar BA Laperriere NJ Bernstein M Jiang H Ménard C Chung C Salvage radiosurgery for brain metastases: prognostic factors to consider in patient selection Int J Radiat Oncol Biol Phys 2014 88 137 142 24331660 10.1016/j.ijrobp.2013.10.003

[B99] Chao ST Barnett GH Vogelbaum MA Angelov L Weil RJ Neyman G Reuther AM Suh JH Salvage stereotactic radiosurgery effectively treats recurrences from whole-brain radiation therapy Cancer 2008 113 2198 2204 18780319 10.1002/cncr.23821

[B100] Caballero JA Sneed PK Lamborn KR Ma L Denduluri S Nakamura JL Barani IJ McDermott MW Prognostic factors for survival in patients treated with stereotactic radiosurgery for recurrent brain metastases after prior whole brain radiotherapy Int J Radiat Oncol Biol Phys 2012 83 303 309 22079723 10.1016/j.ijrobp.2011.06.1987

[B101] Hsu F Kouhestani P Nguyen S Cheung A McKenzie M Ma R Toyota B Nichol A Population-based outcomes of boost versus salvage radiosurgery for brain metastases after whole brain radiotherapy Radiother Oncol 2013 108 128 131 23742960 10.1016/j.radonc.2013.04.025

[B102] Kim DH Schultheiss TE Radany EH Badie B Pezner RD Clinical outcomes of patients treated with a second course of stereotactic radiosurgery for locally or regionally recurrent brain metastases after prior stereotactic radiosurgery J Neurooncol 2013 115 37 43 23813230 10.1007/s11060-013-1191-6

[B103] Barnholtz-Sloan JS Yu C Sloan AE Vengoechea J Wang M Dignam JJ Vogelbaum MA Sperduto PW Mehta MP Machtay M Kattan MW A nomogram for individualized estimation of survival among patients with brain metastasis Neuro Oncol 2012 14 910 918 22544733 10.1093/neuonc/nos087 PMC3379797

[B104] Lester SC Taksler GB Kuremsky JG Lucas JT Jr Ayala-Peacock DN Randolph DM 2nd Bourland JD Laxton AW Tatter SB Chan MD Clinical and economic outcomes of patients with brain metastases based on symptoms: an argument for routine brain screening of those treated with upfront radiosurgery Cancer 2014 120 433 441 24452675 10.1002/cncr.28422 PMC9168957

